# Potential Racial Bias During Pediatric Emergency Care: A Simulation Study

**DOI:** 10.7759/cureus.79843

**Published:** 2025-02-28

**Authors:** Vaishnavi J Patel, Elizabeth Byrne, Jendi Haug, Kellie Williams, Shad Deering

**Affiliations:** 1 Office of Research and Innovation, University of the Incarnate Word School of Osteopathic Medicine, San Antonio, USA; 2 Department of Pediatrics, CHRISTUS Children's/Baylor College of Medicine, San Antonio, USA; 3 Department of Obstetrics and Gynecology, CHRISTUS Children's/Baylor College of Medicine, San Antonio, USA

**Keywords:** healthcare disparities, health disparity, high-fidelity simulation, implicit bias, pediatric emergency department, pediatric emergency medicine, pediatric sepsis, sepsis management, skin color, unconscious bias

## Abstract

Objectives

Despite education to help reduce differences in care, minorities continue to have worse health outcomes. Implicit biases are known to contribute to disparities in healthcare, even in pediatric populations. Prior simulation studies have used computer simulations or standardized patient encounters to understand the potential role of implicit bias in patient interactions. High-fidelity simulation is another method for evaluating patient management decisions in a controlled environment. This study aimed to evaluate whether high-fidelity simulation could reveal differences in pediatric patient management decisions during an emergency based on the skin color of a pediatric manikin.

Methods

A standard simulation scenario was created for a pediatric sepsis case. Nineteen first-year pediatric residents in their first month of internship were offered the opportunity to participate, and informed consent was obtained for our study approved by CHRISTUS Health. Participants were not made aware that the study involved any evaluation related to manikin skin color. Enrolled participants were randomized to run through the scenario with either a dark or light skin manikin with a standardized patient of the same skin tone playing the role of the parent. After simulations were completed, videos were analyzed by physician graders with a standardized evaluation form documenting interventions and timing.

Results

The majority of care was not statistically different for both light- and dark-skinned manikins in the sepsis simulation. However, there were some significant differences noted. All of the dark-skinned infants received oxygen while only 55.5% (5/9) of the light-skinned infants received oxygen (p = 0.03). Additionally, 89% (8/9) of the light-skinned infants received compressions after asystole occurred while only 40% (4/10) of the dark-skinned infants received compressions (p = 0.05).

Discussion

In a simulated pediatric sepsis case, there were significant differences in some interventions on the basis of skin color. While we can only theorize about the reason for these, they may have resulted from assumptions of different etiologies of the emergencies or unconscious reactions to the color of the manikin. Simulation is a unique approach to evaluate this important topic using standardized simulations and evaluation tools, and we intend to use these results to hone further research investigating reasons for the identified differences.

## Introduction

There has been much attention given to the healthcare inequalities between different races. Despite educational initiatives to mitigate implicit bias in healthcare intended to help reduce these disparities, minorities continue to have worse outcomes [1]. Unfortunately, these disparities are not limited to adults and have also been documented in the pediatric population [2]. For example, minority pediatric trauma patients have longer emergency department (ED) wait times, prolonged ED lengths of stay, and decreased prescriptions for analgesia at discharge [3]. Black and Hispanic patients diagnosed with appendicitis are more likely to have perforations while Black patients are more prone to have delays to surgery, less likely to have laparoscopic surgery, and more likely to have longer lengths of stay compared to White patients [4]. Additionally, Black pediatric patients with severe pain are less likely to receive opioid analgesia and those with moderate pain are less likely to receive any analgesic compared to White patients [5]. Although several studies have demonstrated these differences, little is known of the exact cause of the disparity or how to address it though it has been postulated that a potential reason for the differences in care may be related to the implicit bias of the healthcare professional.

Simulation-based medical education is a tool that can be used to educate learners about medical decision-making, resuscitation, and communication [6-8]. Previous simulation studies have used computer-simulated or standardized patient encounters to better understand the role of implicit bias by healthcare workers in patient interactions with minority populations [9,10]. In these studies, the providers’ implicit bias was measured using the validated implicit association test to measure racial biases [11]. It is not known, however, exactly how the implicit biases identified may affect actual patient care in the pediatric patient population. To our knowledge, no prior studies have evaluated the effect of implicit bias on the treatment of pediatric patients in emergent clinical situations. In order to evaluate this, we undertook this study with a high-fidelity emergent pediatric simulation scenario in order to evaluate and characterize the effect of possible implicit biases in the context of racial differences. We hypothesized that underlying implicit biases may alter the care given to patients of dark skin tones. We hope that identifying these disparities may allow for the design of future interventions to mitigate those differences.

## Materials and methods

**Figure 1:**
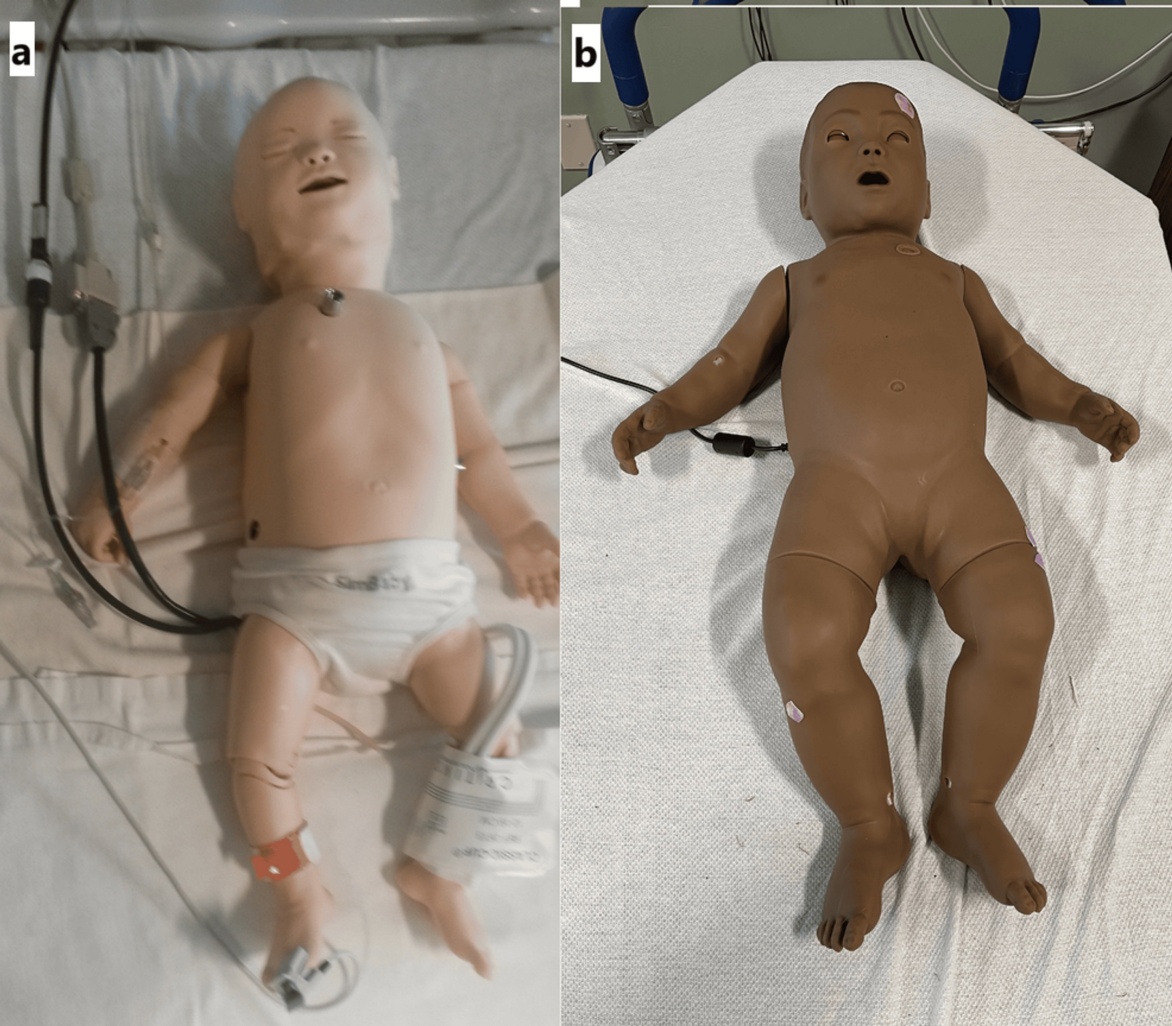
Simulation Models Laerdal Medical Corporation White SimBaby Classic (a) and Laerdal Medical Corporation Dark SimBaby (b). These were the infant manikins used in our study.

With support from the pediatric residency program directors of two local programs, our team presented the opportunity for first-year pediatric residents in their first month of training to participate in this study approved by CHRISTUS Health (011402). All first-year pediatric residents in this residency program were offered the opportunity to participate in these simulations. From these, 19 residents were recruited prior to one of their scheduled simulation education days to participate in the study and gave written informed consent. Each resident was assigned a study number and randomized into light-skinned manikin and dark-skinned manikin groups. They then participated in a pediatric case of a critically ill sepsis resuscitation. Participants were not informed that the study involved an evaluation related to manikin skin color. Each first-year pediatric resident went through one individual simulation; therefore, 19 simulations were completed in total. The simulation models that were used were from Laerdal Medical Corporation (Stavanger, Norway); the White SimBaby Classic and the Dark SimBaby 2019 models can be seen in Figure 1.

Simulation scenarios and evaluation forms were developed based on current best practices and national Pediatric Advanced Life Support (PALS) standards in the United States of America. Standardized patients were hired and trained to portray the parent of the infant in each scenario, and we ensured they were matched in skin tone with the infant in each scenario. Additionally, an embedded participant played the role of the nurse for each scenario. The Standardized patients and embedded participants were given scripts for the scenarios with identical answers. They were trained for the simulation scenario by one of the physician investigators. Additionally, the scenario was piloted with senior residents prior to implementation. All simulation scenarios were video-recorded for evaluation.

For the infant sepsis resuscitation scenario, performance was evaluated with components contained within the PALS-based checklist. In addition to noting if specific interventions were performed, we documented the time to initiate appropriate resuscitative measures, such as chest compressions, epinephrine doses, and fluid boluses. The checklist was created and validated by physician evaluators. We also noted the time it took to administer pain medication to the infant, what medication was ordered, and the dose given. For the purposes of this scenario, the patient had a pain score of 7 based on the Wong-Baker Faces Pain Severity Score, and this was modeled based on the standardized patient’s scripted responses. At the completion of the simulation, the evaluator asked the resident to state what their top 3 differential diagnoses were in order of what they thought was the most likely underlying cause for the clinical situation. After the simulations were concluded, all participants were debriefed on their medical care of the case.

With the completion of all simulation sessions, study personnel reviewed the resident simulation videos and utilized the evaluation checklist to record performance and measure time intervals. Each video was reviewed by two separate physician expert personnel. For any items with discordant answers, videos were reviewed, and group consensus was obtained by the physician experts in order to ensure consistent and accurate reporting of results and data. Study numbers and evaluation form data for all residents were entered into an electronic database with no plans for linkage data.

Statistical analysis was performed using Microsoft Excel statistical software (Microsoft Corp., Redmond, WA, US). For continuous variables, an F-test for variances was performed to determine whether a t-test assuming equal or unequal variances was ideal. A p-value of greater than 0.05 indicated equality of variances, for which a t-test was performed. A p-value of less than 0.05 indicated unequal variances, for which a Welch t-test and Wilcoxon Mann-Whitney test were performed. For dichotomous variables, two-tailed Fisher's exact test was performed. A p-value of less than 0.05 was considered significant. If a variable had a p-value that was greater than 0.05 but less than 0.1, a post hoc power analysis was performed to determine whether the sample size was adequate to find a statistically significant result. All methodology and statistical analyses in this study were independently reviewed and approved by a third-party statistician.

## Results

Nineteen first-year pediatric residents agreed to participate in the study. Overall, most of the care was not statistically different for both light- and dark-skinned manikins. However, there were some significant differences noted as can be seen in Tables 1, 2.

**Table 1:** Differences in the Performance of Interventions Based on the Skin Color of Infants The summary data is represented by the number outside the parentheses and the percentage within the parentheses. All of these variables are dichotomous, reporting whether the intervention was performed or not. IO: intraosseous; BVM: bag-valve-mask

	Light-skinned (n = 9)	Dark-skinned (n = 10)	Fisher's exact p-value
Leads placed	8 (88.88%)	10 (100%)	0.474
Vital signs reviewed	9 (100%)	8 (80%)	0.474
IO ordered	3 (33.33%)	6 (60%)	0.370
1st fluid bolus	8 (88.88%)	6 (60%)	0.303
Antibiotics started	2 (22.22%)	0 (0%)	0.211
Oxygen placed	5 (55.55%)	10 (100%)	0.033
Compressions started	8 (88.88%)	4 (40%)	0.057
BVM ventilation	7 (77.77%)	7 (70%)	1.000
Epinephrine started	8 (88.88%)	7 (70%)	0.582
Top differential-sepsis	3 (33.33%)	2 (20%)	0.628
Top 3 differential-sepsis	6 (66.67%)	2 (20%)	0.070

**Table 2:** Differences in the Time of Interventions Based on the Skin Color of Infants The mean is outside the parentheses in the summary data, and the standard deviation is within the parentheses. The units are in seconds for all variables in this table. All reported times are measured from the beginning of care, with the exception of epinephrine, which was recorded from the initiation of compressions. IO: intraosseous

	Light-skinned (n = 9)	Dark-skinned (n = 10)	t-test p-value	Welch's t-test p-value	Wilcoxon Mann-Whitney test p-value
Time to lead placement	98.75 (71.49)	132.2 (146.28)		0.536	0.965
Time to IO ordered	360 (144.22)	456.83 (53.02)		0.377	0.366
Time to 1st fluid bolus	342.75 (173.64)	371.83 (190.68)	0.771		
Time to oxygen placement	551.2 (282.15)	326.4 (172.6)		0.075	0.098
Time to epinephrine	90 (40.35)	77.29 (37.66)	0.541		

**Figure 2:**
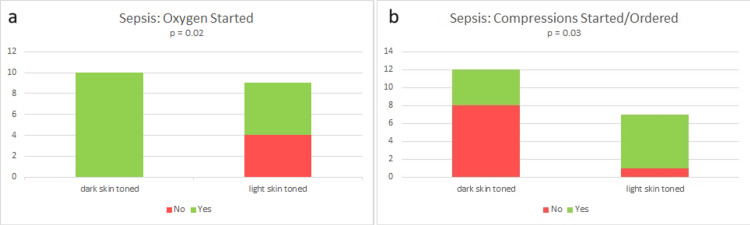
(a and b) Oxygen and Compressions Started in Sepsis

Significant differences were observed in the simulations, with 100% (10/10) of dark-skinned infant manikins receiving oxygen compared to only 55% (5/9) of light-skinned infant manikins during the sepsis simulation (p = 0.033). Additionally, 88.9% (8/9) of the light-skinned infant manikins received chest compressions after asystole occurred while only 40% (4/10) of the dark-skinned infant manikins did (p = 0.057). These findings can be visualized in Figure 2.

We additionally reviewed the differential diagnoses that the participants reported after the simulations and compared those involving the light- and dark-skinned infant manikins. We noted that 66.7% (6/9) of the time simulations utilizing light-skinned infant manikins had sepsis among the top 3 differential diagnoses considered by the first-year pediatric resident. On the other hand, only 20% (2/10) of dark-skinned infant manikin simulations had sepsis listed as a possible diagnosis (p = 0.070). In addition, there was a significant difference in residents having either their top diagnosis (p = 0.030) or one of their top 3 diagnoses (p = 0.003) be related to respiratory problems such as respiratory arrest, acute respiratory distress syndrome (ARDS), asthma, and pneumonia in the dark-skinned as compared to simulations with light-skinned infant manikins. The average time to start oxygen had a p-value that approached significance as it was 326.4 seconds for the dark-skinned infant manikins and 551.2 seconds for the light-skinned infant manikins (p = 0.075).

**Figure 3:**
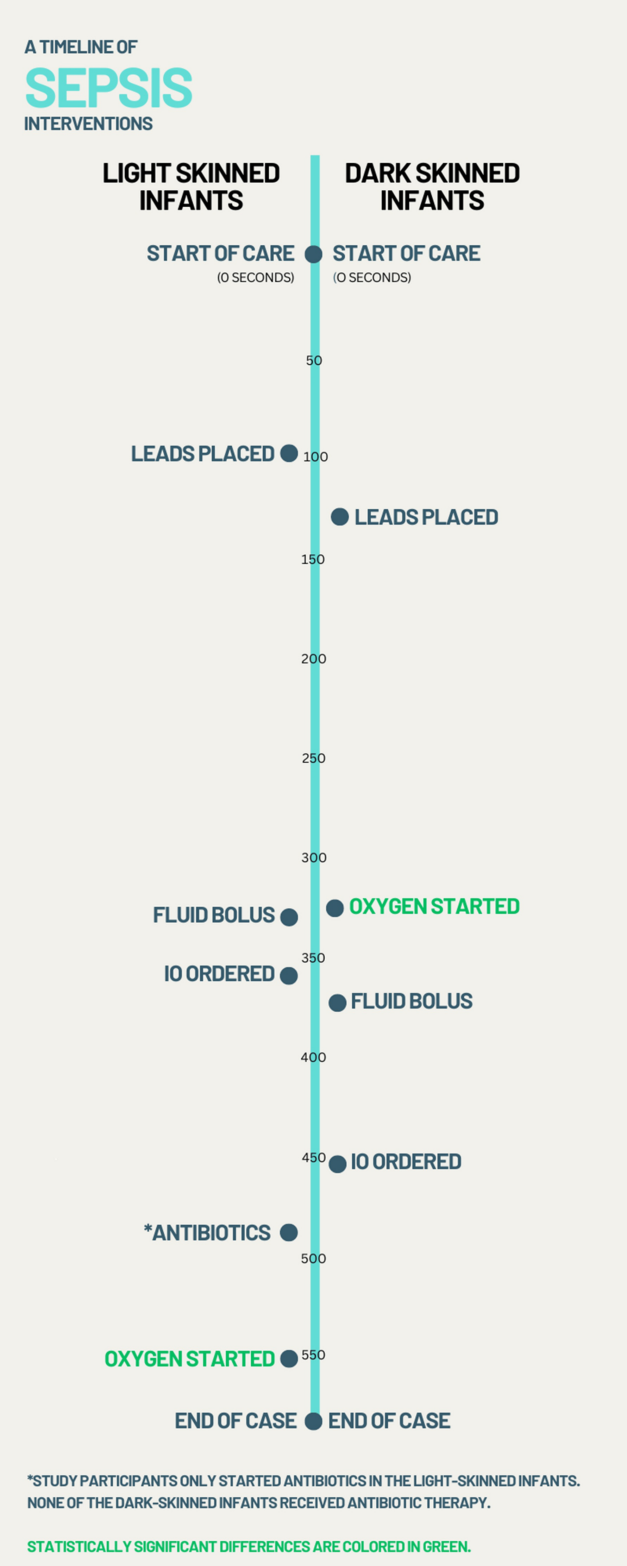
Timeline of Pediatric Sepsis Interventions This timeline is presented in seconds from the start of care of the infant during sepsis protocols to the point where no other significant interventions were performed. Antibiotics are not listed on the dark-skinned infant manikin side of the diagram as there were no antibiotics ordered for this group during any of the scenarios. The left side of the timeline is the average scenario time for interventions for light-skinned infant manikins while the right side of the timeline is the average scenario time for dark-skinned infant manikins. IO: intraosseous

For many of the other interventions measured, although differences were not statistically significant, there was a pattern present where the timing of them was delayed in the dark-skinned infant manikins. The exceptions to this pattern include oxygen, which was started earlier in the dark-skinned infant manikins, and antibiotics, which were only started in light-skinned infant manikins as seen in Figure 3.

## Discussion

In our study of a simulated pediatric sepsis case, there were significant differences in some interventions between first-year pediatric residents in the care provided to dark and light skin tone infant simulators and in the differential diagnoses despite nearly identical scenarios. While we can only theorize the reason for these differences, it may have resulted from assumptions regarding the etiology of the emergency or their unconscious reactions to the color of the manikins. It is important to highlight that, despite extensive literature documenting worse outcomes among Black patients, relatively few differences were observed between the dark-skinned and light-skinned infant manikins in this study.

We found that based on an average of all the participants in this study, oxygen was the only intervention that was given earlier in dark-skinned infants than light-skinned infants and every other intervention occurred later. However, since the dark-skinned infants were significantly more likely to receive a diagnosis associated with a respiratory issue, this may explain the reason for the difference noted in oxygen being provided. And, when the timeline was limited to residents who listed sepsis as their top differential diagnosis, oxygen placement was delayed in the dark-skinned infants on average (390.5 seconds) compared to light-skinned infants (268.5 seconds) (p-value = 0.40). However, even for the two residents who put sepsis as their top differential diagnosis for a dark-skinned infant, neither of them ordered antibiotics or a fluid bolus during the scenario.

However, even noting the different assumptions about the underlying etiology cannot explain the trend observed in compressions between the two groups of infants after asystole. In reviewing the cases, compressions were started in 88.8% of light-skinned infants compared to only 40% of dark-skinned ones (p = 0.057). Even if there was a bias related to the underlying etiology of the presentation, such a key intervention as compressions after asystole should not have been delayed.

It is worth noting that all our participants were new in their medical training as pediatric residents. There are studies that demonstrate increasing prejudice with age, but these have not been performed on healthcare providers specifically [12]. However, there are also studies showing that healthcare providers are just as prejudiced as the general population [13]. Other studies have shown a statistically significant level of prejudiced beliefs and practices among healthcare providers [14].

We recognize that our study has several limitations. For example, our analysis was limited by a small sample size, which may have reduced the ability to detect differences in care. The small sample size may also mean that findings could represent a difference in skill between dark- and light-skinned manikin simulation residents. Since resident demographics were not recorded as a part of this study, it is possible that resident age and race may play a confounding role. Additionally, no matter the fidelity of the simulator, it will have some limits on its ability to capture real-life differences in care and skin color. The first-year pediatric residents may also have been aware of the issue of health disparities due to educational offerings during their training and made conscious efforts to avoid any disparities in their care. Our study did not consider systemic barriers such as insurance coverage, language barriers, and issues of access that may play into real-life treatment plans, but there may be preconceived notions of these aspects of care in the participants that impacted their care.

There are many theories in the literature for why interventions given by healthcare providers may vary based on skin color, and it is well documented that there are disparities in healthcare outcomes between individuals of different races. Providers may have held preconceived notions or stereotypes regarding the health of darker-skinned infants, potentially perceiving their need for care as less urgent compared to their light-skinned counterparts. The issue of healthcare disparities among different racial groups has been extensively studied, yet these inequities persist, particularly among minority populations, even with ongoing educational efforts aimed at addressing them [1]. Importantly, these disparities extend beyond adults and are prevalent in pediatric care as well [2]. For instance, minority pediatric trauma patients often face longer ED wait times and extended ED stays and are less likely to receive adequate analgesia at discharge [3]. Black and Hispanic children with appendicitis are disproportionately affected, with higher rates of perforation; Black children, in particular, are more likely to experience surgical delays, are less likely to undergo laparoscopic surgery, and are more likely to have longer hospital stays compared to their White counterparts [4]. Furthermore, Black pediatric patients with severe pain are less likely to receive opioid analgesics, while those with moderate pain are less likely to receive any form of pain relief [5]. Although these differences in care are well documented, the underlying causes remain poorly understood. Implicit bias among healthcare providers has been suggested as a potential contributing factor, but further investigation is needed to elucidate its role and develop effective interventions. While implicit bias could contribute to delays in the care of darker-skinned infants in pediatric emergency situations, this study did not investigate this aspect in sufficient depth to confirm or refute such a connection. Bias may affect how quickly a healthcare provider responds to an emergency, including delays in administering life-saving interventions. Regardless of the cause, the finding highlights the need for ongoing efforts to address healthcare disparities and ensure that all patients receive high-quality, competent care.

## Conclusions

Simulation presents a valuable tool for exploring these disparities, offering standardized scenarios and evaluations with new pediatric trainees. Although limited by a small sample size, our findings serve as a foundation for future research to determine whether additional disparities emerge as providers advance in training. While numerous disparities in care along racial and ethnic lines have been documented in the literature, our simulation-based study on the management of a septic infant identified only two significant differences between scenarios involving light-skinned and dark-skinned manikins. It is challenging to attribute these differences solely to racism or implicit bias. These results underscore the complexity of factors contributing to racial and ethnic disparities in healthcare and emphasize the need for ongoing efforts to achieve equity in pediatric care. This study highlights the critical need for the healthcare community to recognize and address implicit biases to ensure equitable care for all infants, irrespective of skin tone.
